# Synthesis, structural characterization, Hirshfeld surface analysis and QTAIM analysis of 3-(4-cyano­thio­phen-3-yl)-[1,2,4]selena­diazolo[4,5-*a*]pyridin-4-ium chloride

**DOI:** 10.1107/S205698902500115X

**Published:** 2025-02-14

**Authors:** Alexander A. Sapronov, Evgeny A. Dukhnovsky, Alexey S. Kubasov, Alexander S. Novikov, Maria M. Grishina, Ekaterina V. Dobrokhotova, Milena R. Komarovskikh, Namiq Q. Shikhaliyev, Mehmet Akkurt, Ajaya Bhattarai, Alexander G. Tskhovrebov

**Affiliations:** a Peoples’ Friendship University of Russia, 6 Miklukho-Maklaya Street, Moscow, 117198, Russia; b Kurnakov Institute of General and Inorganic Chemistry, Russian Academy of Sciences, Leninsky Prosp. 31, 119071 Moscow, Russia; c Institute of Chemistry, Saint Petersburg State University, Universitetskaya Nab. 7/9, 199034 Saint Petersburg, Russia; d Department of Chemical Engineering, Baku Engineering University, Hasan Aliyev Street 120, Baku AZ0101, Azerbaijan; e Department of Physics, Faculty of Sciences, Erciyes University, 38039 Kayseri, Türkiye; f Department of Chemistry, M.M.A.M.C (Tribhuvan University) Biratnagar, Nepal; g Research Institute of Chemistry, Peoples’ Friendship University of Russia, Miklukho-Maklaya St., 6, Moscow 117198, Russian Federation; University of Neuchâtel, Switzerland

**Keywords:** crystal structure, chalcogen-hydrogen bonding, 1,2,4-seleno­diazole, Hirshfeld surface analysis

## Abstract

The title compound, C_11_H_6_N_3_SSe^+^·Cl^−^, produced by the reaction between 3,4-di­cyano­thio­phene and 2-pyridyl­selenyl chloride was isolated as a salt that crystallizes in the triclinic space group *P*1. Notable features include strong chalcogen inter­actions (Se⋯Cl and Se⋯S), as revealed through Hirshfeld surface analysis, which also highlights significant contributions from N⋯H/H⋯N, C⋯H/H⋯C and H⋯H contacts in the crystal packing. Supra­molecular inter­actions were further analysed using density functional theory (DFT) and quantum theory of atoms in mol­ecules (QTAIM) at the ωB97XD/6–311++G** level of theory.

## Abstract 2

The title compound was produced by the reaction between 3,4-di­cyano­thio­phene and 2-pyridyl­selenyl chloride and isolated as a salt that crystallizes in the triclinic space group *P*1. Notable features include strong chalcogen inter­actions (Se⋯Cl and Se⋯S), as revealed through Hirshfeld surface analysis, which also highlights significant contributions from N⋯H/H⋯N, C⋯H/H⋯C and H⋯H contacts in the crystal packing.

## 1. Chemical context

Recently, we discovered that 2-pyridyl­selenyl reagents undergo cyclization with unactivated nitriles under mild conditions, enabling the synthesis of previously unknown 1,2,4-selena­diazo­les (Khrustalev *et al.*, 2021 ▸). The presence of two σ-holes on the selenium atom imparts a unique property to 1,2,4-selena­diazo­les, allowing them to form supra­molecular dimers *via* four-centre Se_2_N_2_ chalcogen bonds (Grudova *et al.*, 2022 ▸). Additionally, we explored their cyclo­addition reactions with various nucleophilic mol­ecules, demonstrating the versatility of 2-pyridyl­selenenyl reagents (Artemjev *et al.*, 2022 ▸, 2024 ▸; Sapronov *et al.*, 2022 ▸, 2024 ▸). In this report, we describe the structure of 3-(4-cyano­thio­phen-3-yl)-[1,2,4]selena­diazolo[4,5-*a*]pyridin-4-ium chloride, which was obtained from the reaction between 3,4-di­cyano­thio­phene and 2-pyridyl­selenenyl.
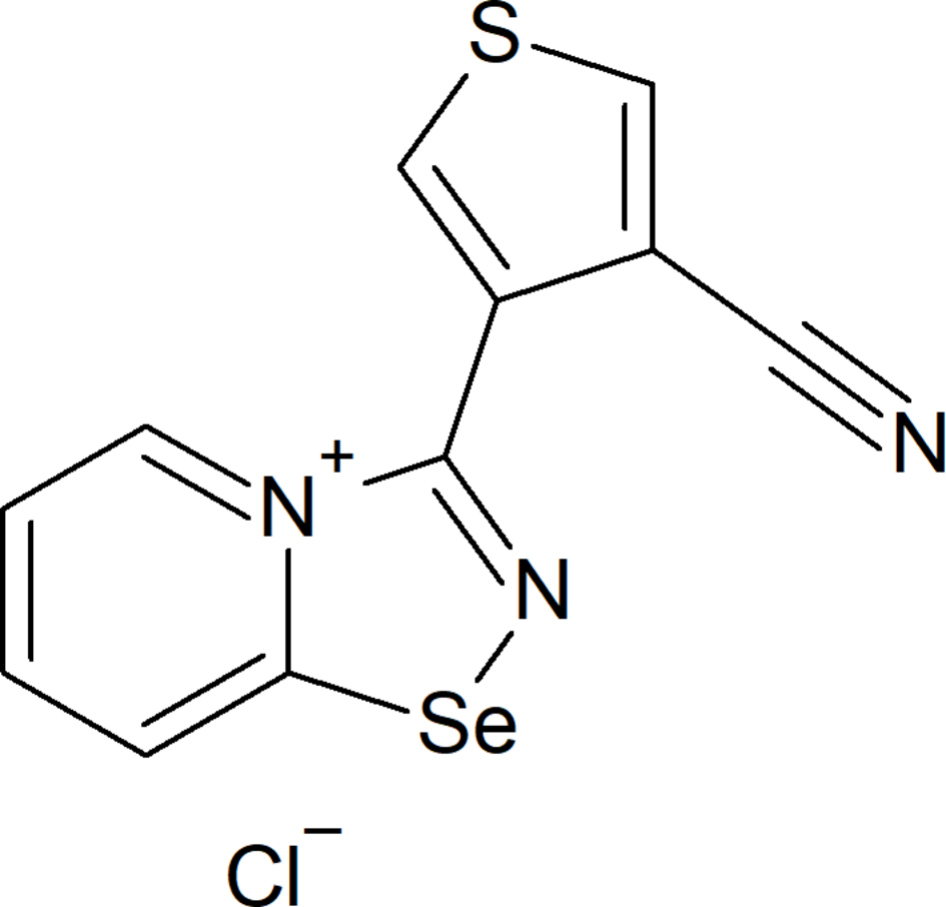


## 2. Structural commentary

**Figure 1:**
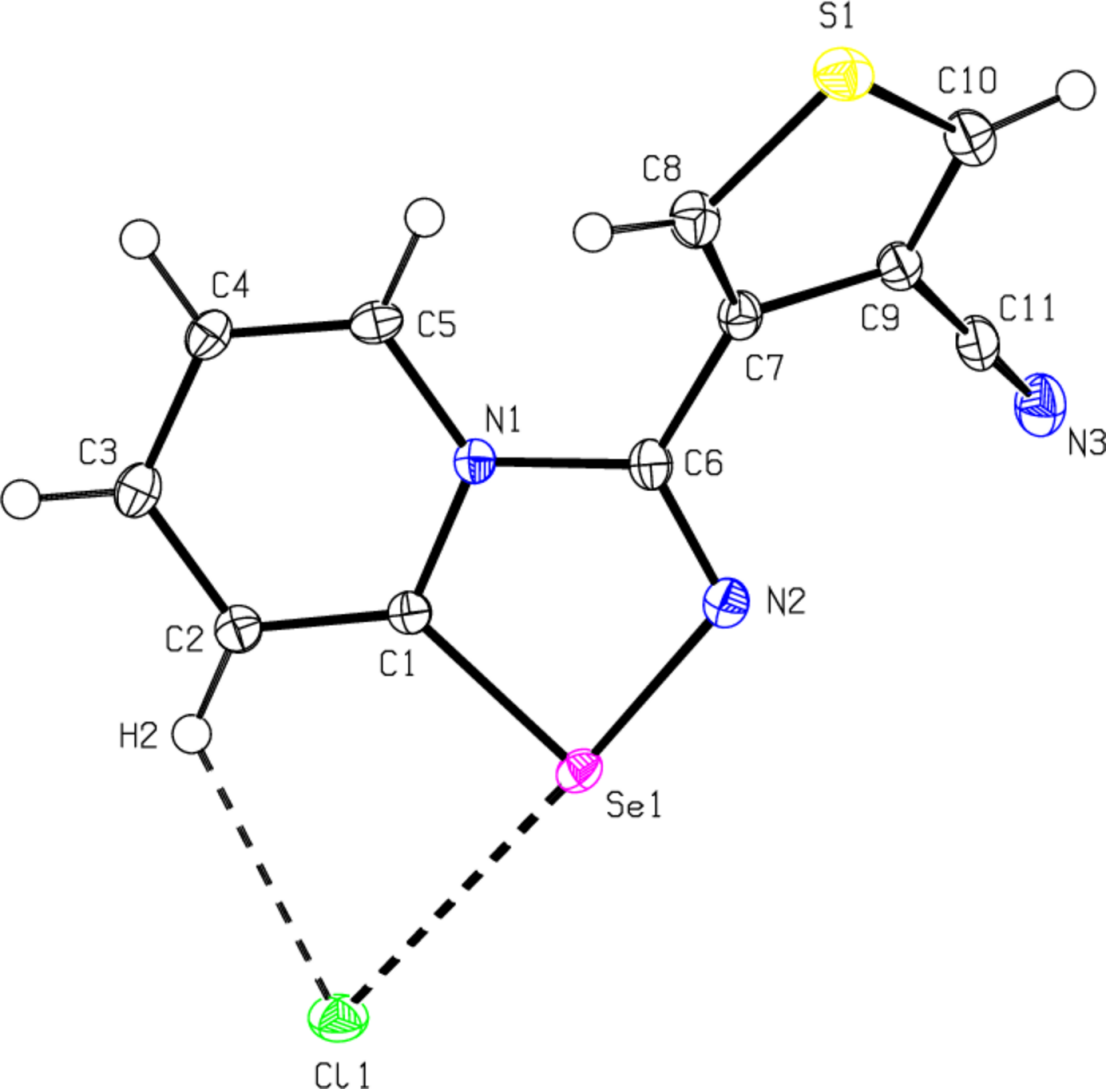
Mol­ecular structure of 3-(4-cyano­thio­phen-3-yl)-[1,2,4]selena­diazolo[4,5-*a*]pyridin-4-ium chloride. Displacement ellipsoids are drawn at the 50% probability level.

As shown in Fig. 1 ▸, the nine-membered ring system (Se1/N1/N2/C1–C6) of the cation is essentially planar [the maximum deviation is 0.034 (2) Å for C6] and makes an angle of 47.40 (9)° with the least-squares plane of the thio­phene ring (S1/C7–C10). The intra­molecular inter­action between the Cl^−^ anion and the Se1 and (C2)H2 atoms of the cation forms an *S*(5) ring motif and thus the title mol­ecule has a stable conformation. The Se1—C1 and Se1—N2 bond lengths are 1.865 (2) and 1.8511 (19) Å, respectively. The lengths of the single C6—N1 bond and the double C6—N2 bond are 1.426 (3) and 1.283 (3) Å, respectively. The bond length and angle values are comparable to those of similar compounds (see *Database survey* section).

## 3. Supra­molecular features

**Figure 2:**
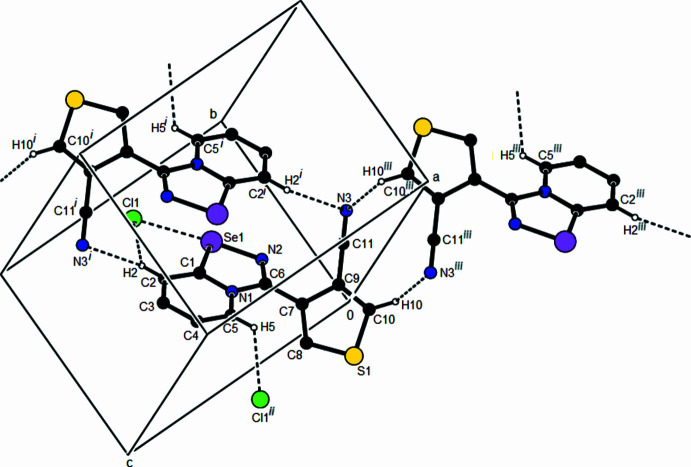
A partial packing diagram showing the C—H⋯N and C—H⋯Cl hydrogen bonds (dashed lines). H atoms not involved in these inter­actions have been omitted for clarity. Symmetry codes: (i) −*x* + 1, −*y* + 1, −*z* + 1; (ii) *x*, *y* − 1, *z*; (iii) −*x* + 1, −*y*, −*z*.

**Figure 3:**
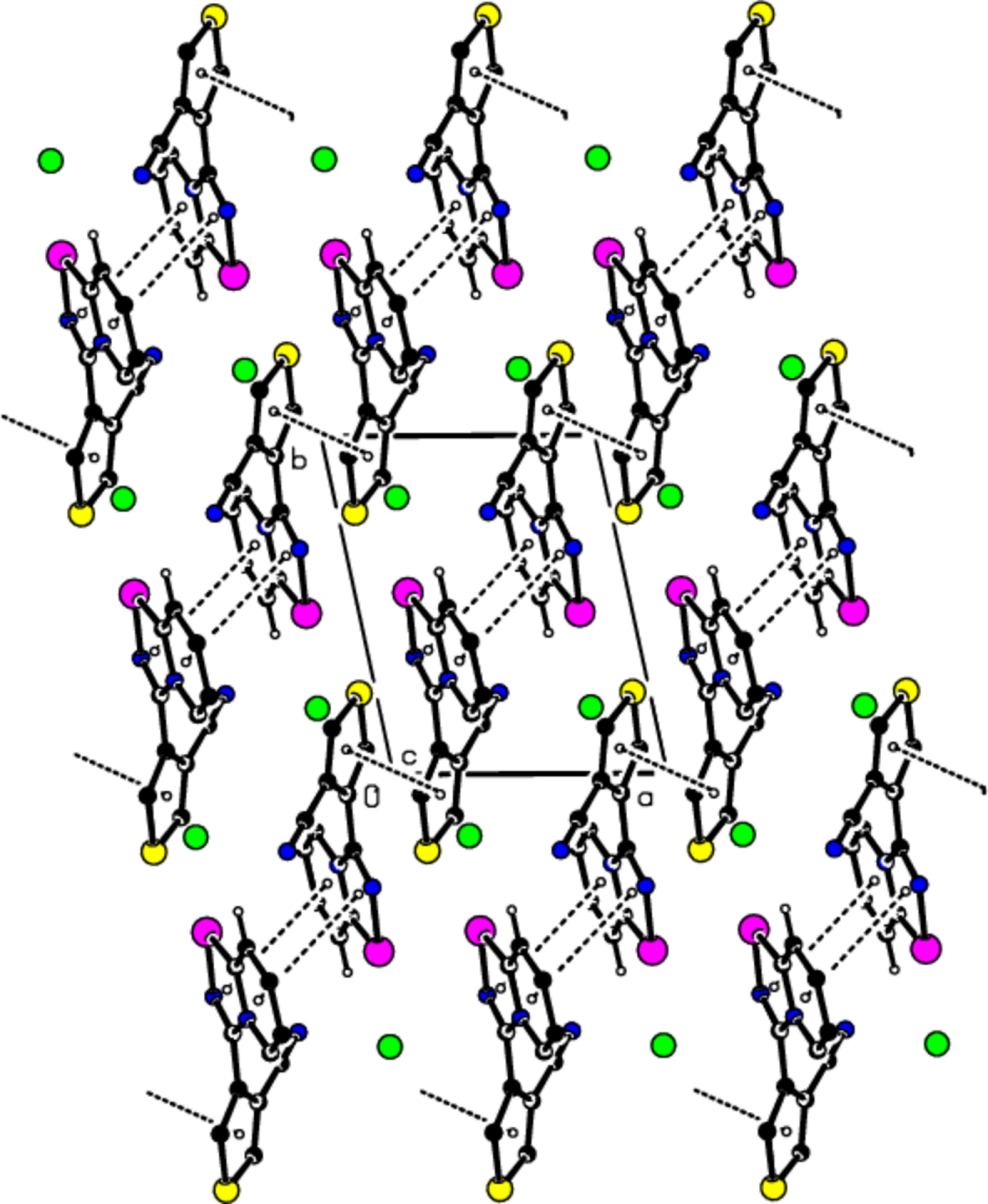
Crystal packing showing the π-π- stacking inter­actions between adjacent cations (dashed lines).

In the crystal, pairs of cations are linked by C10—H10⋯N3 hydrogen bonds, thus forming a dimeric 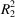
(10) ring motif (Table 1 ▸; Fig. 2 ▸; Bernstein *et al.*, 1995 ▸). These dimers are connected by pairs of C2—H2⋯N3 hydrogen bonds, forming inversion dimers with 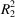
(18) ring motifs, which lead to the formation of ribbons propagating along the *c*-axis direction (Table 1 ▸). There are π–π stacking inter­actions between the rings of the bicyclic ring systems of two adjacent cations [Fig. 3 ▸; *Cg*1⋯*Cg*3^i^ = 3.964 (2) Å, slippage = 1.955 Å; *Cg*3⋯*Cg*1^i^ = 3.964 (2) Å, slippage = 1.851 Å; symmetry code: (i) −*x* + 1, −*y* + 1, −*z* + 1; *Cg*1 and *Cg*3 are the centroids of the Se1/N2/C6/N1/C1 and N1/C1–C5 rings, respectively], as well as between two thio­phene groups (Fig. 3 ▸). The distance between the centroids (*Cg*2 and *Cg*2^iv^) of the thio­phene rings (S1/C7–C10) is 3.849 (2) Å [slippage = 1.831 Å; symmetry code: (iv) −*x*, −*y*, −*z*]. These π–π stacking inter­actions between thio­phene rings form ribbons along the [110] direction. Overall, the crystal is consolidated by this three-dimensional network formed by π-π stacking inter­actions and inter­molecular C—H⋯N inter­actions.

## 4. Hirshfeld surface analysis

**Figure 4:**
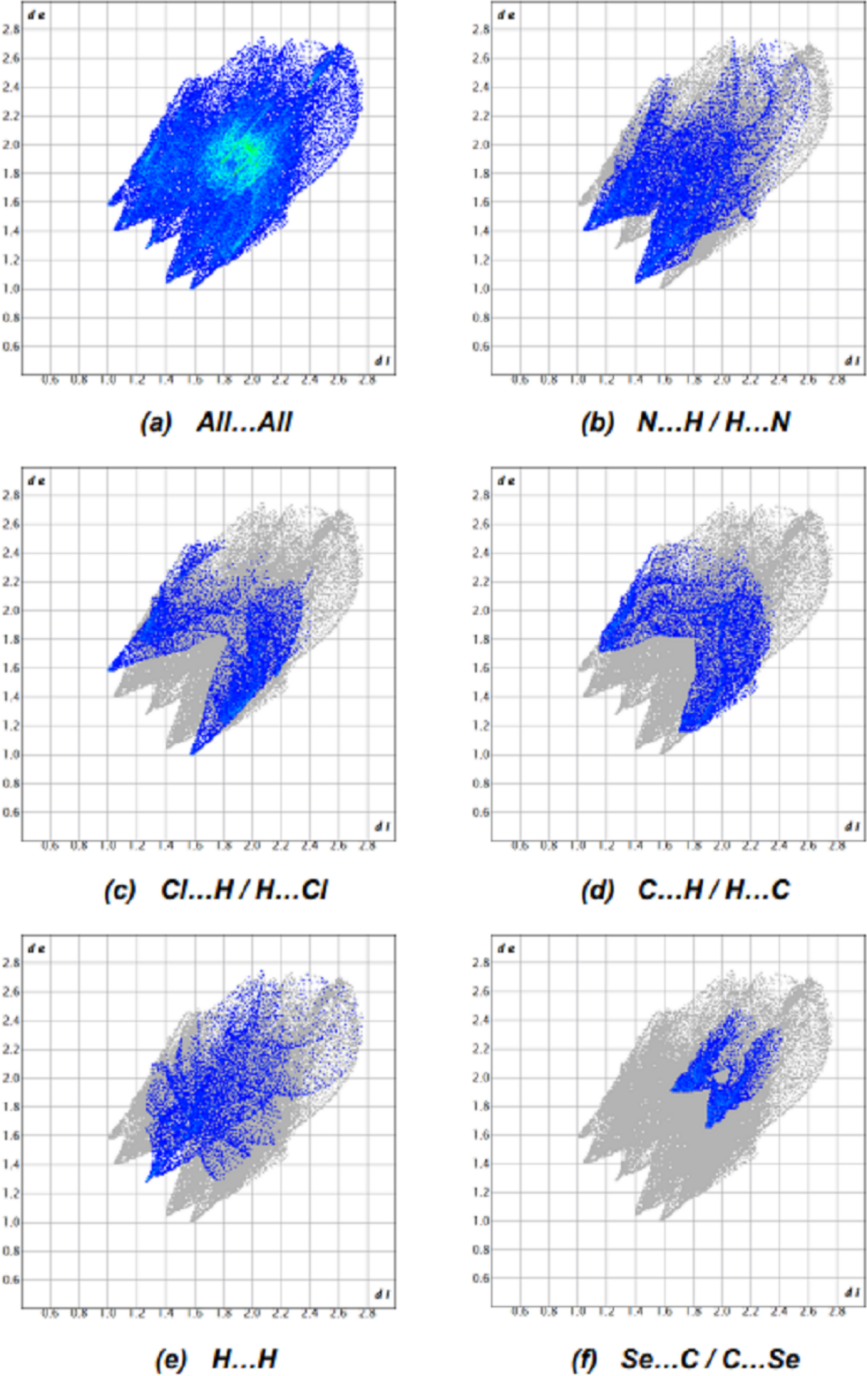
The two-dimensional fingerprint plots, showing (*a*) all inter­actions, and those delineated into (*b*) N⋯H/H⋯N, (*c*) Cl⋯H/H⋯Cl, (*d*) C⋯H/H⋯C, (*e*) H⋯H and (*f*) Se⋯C/C⋯Se inter­actions; *d*_e_ and *d*_i_ represent the distances from a point on the Hirshfeld surface to the nearest atoms outside (external) and inside (inter­nal) the surface, respectively.

In order to qu­antify the inter­molecular inter­actions in the crystal, *Crystal Explorer 17.5* (Spackman *et al.*, 2021 ▸) was used to generate Hirshfeld surfaces and two-dimensional fingerprint plots (Fig. 4 ▸). The most important inter­atomic contact is N⋯H/H⋯N as it makes the highest contribution to the crystal packing (22.2%, Fig. 4 ▸*b*). The other major contributors are the Cl⋯H/H⋯Cl (13.4%, Fig. 4 ▸*c*), C⋯H/H⋯C (12.4%, Fig. 4 ▸*d*) and H⋯H (11.3%, Fig. 4 ▸*e*) inter­actions. Other, smaller contributions (Table 2 ▸) are made by Se⋯C/C⋯Se (6.9%, Fig. 4 ▸*f*), Cl⋯C/C⋯Cl (5.3%), S⋯H/H⋯S (4.8%), S⋯N/N⋯S (4.4%), C⋯C (3.7%), Se⋯S/S⋯Se (3.6%), S⋯C/C⋯S (3.1%), Se⋯H/H⋯Se (2.4%), Cl⋯N/N⋯Cl (2.3%), C⋯N/N⋯C (2.2%), S⋯S (1.0%), Se⋯N/N⋯Se (0.9%) and Se⋯Cl/Cl⋯Se (0.1%) inter­actions.

## 5. QTAIM analysis

**Figure 5:**
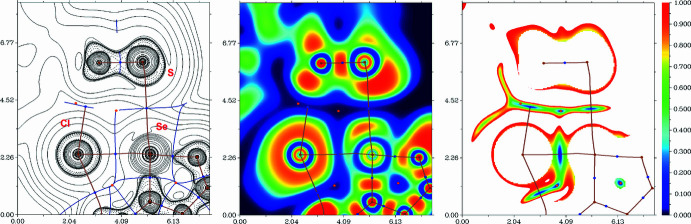
Contour line diagram of the Laplacian of electron density distribution ∇^2^ρ(**r**), bond paths, and selected zero-flux surfaces (left panel), visualization of electron localization function (ELF, centre panel) and reduced density gradient (RDG, right panel) analyses for chalcogen bonds Se⋯Cl and Se⋯S. Bond critical points (3, −1) are shown in blue, nuclear critical points (3, −3) in pale brown and ring critical points (3, +1) in orange. Bond paths are shown as pale-brown lines, length are in Å, and the colour scale for the ELF and RDG maps is presented in a.u.

Inspection of the crystallographic data reveals the presence of Se⋯Cl and Se⋯S chalcogen bonds as being the most non-trivial non-covalent inter­actions. To better understand the nature and approximately qu­antify the strength of these inter­molecular contacts, DFT calculations followed by topological analysis of the electron density distribution (QTAIM analysis) were carried out at the ωB97XD/6-311++G** level of theory. Results of the QTAIM analysis for chalcogen bonds Se⋯Cl and Se⋯S are summarized in Table 3 ▸; the contour line diagram of the Laplacian of electron density distribution ∇^2^ρ(**r**), bond paths, and selected zero-flux surfaces, visualization of the electron localization function (ELF) and reduced density gradient (RDG) analyses for these non-covalent contacts are shown in Fig. 5 ▸.

The QTAIM analysis of the model supra­molecular associate demonstrates the presence of bond critical points (3, −1) for chalcogen bonds Se⋯Cl and Se⋯S (Table 3 ▸ and Fig. 5 ▸). The low magnitude of the electron density, the positive values of the Laplacian of electron density, very close to zero values of energy density, magnitudes of the electron localization function in these bond critical points (3, −1) and the estimated strengths for appropriate short contacts are typical for chalcogen bonds (Khrustalev *et al.*, 2021 ▸; Mikherdov *et al.*, 2016 ▸, 2018 ▸). The balance between the Lagrangian kinetic energy *G*(**r**) and potential energy density *V*(**r**) in bond critical points (3, −1) for chalcogen bonds Se⋯Cl and Se⋯S reveals that Se⋯S contacts are purely non-covalent, whereas Se⋯Cl contacts have small covalent contribution, (Espinosa *et al.*, 2002 ▸) and the sign of λ_2_ allows these chalcogen bonds to be designated as bonding (attractive, λ_2_ < 0) inter­actions (Johnson *et al.*, 2010 ▸; Contreras-García *et al.*, 2011 ▸).

## 6. Database survey

A search in the Cambridge Structural Database (CSD, Version 5.43, update of September 2022; Groom *et al.*, 2016 ▸) gave only 17 hits for 1,2,4-seleno­diazo­lium salts. The most relevant salts are EHAPUC (Temesgen *et al.*, 2024 ▸), BEYHEW, BEYHIA, BEYHOG, BEYHUM, BEYJAU, BEYJEY, BEYJIC, BEYJOI and BEYJUO (Sapronov *et al.*, 2022 ▸). The mol­ecules of EHAPUC are packed in layers parallel to the *ac* plane. Each row of 1,2,4-seleno­diazo­lium salts in the layer is located anti­parallel to the adjacent one. In addition to Se⋯Cl contacts, the anions form C—H⋯Cl contacts that link the cations and anions both within the layers and between them. BEYHEW, BEYHIA, BEYHOG, BEYHUM, BEYJAU, BEYJEY, BEYJIC, BEYJOI and BEYJUO promote the formation of self-assembled dimers with the recurrent Se_2_N_2_ supra­molecular motif. The dimers are further consolidated by two symmetry-equivalent selenium–arene chalcogen-bond inter­actions.

## 7. Synthesis and crystallization

2-Pyridyl­selenyl chloride was synthesized by a published method (Artemjev *et al.*, 2023 ▸; Khrustalev *et al.*, 2021 ▸). A solution of PhICl_2_ (26 mg, 96 µmol) in CH_2_Cl_2_ (2 mL) was added to a solution of 2,2′-di­pyridyl­diselenide (30 mg, 96 µmol) and thio­phene-3,4-dicarbo­nitrile (13 mg, 96 µmol) in CH_2_Cl_2_ (2 mL), and the reaction mixture was left without stirring at room temperature for 12 h. After that, the solution was deca­nted to leave a yellow precipitate. The solid was washed with Et_2_O (3 × 1 mL) and dried under vacuum. Yield: 40 mg (65%). ^1^H NMR (700 MHz, D_2_O) δ 9.43 (*d*, *J* = 6.8 Hz, 1H), 8.91 (*d*, *J* = 8.7 Hz, 1H), 8.67 (*d*, *J* = 3.0 Hz, 1H), 8.48–8.45 (*m*, 1H), 8.39 (*d*, *J* = 3.0 Hz, 1H), 8.01 (*dd*, *J* = 7.7, 6.5 Hz, 1H). ^13^C NMR (176 MHz, D_2_O) δ 168.4, 149.7, 141.0, 140.0, 136.8, 133.4, 127.5, 126.0, 123.3, 114.0, 110.6.

## 8. Refinement

Crystal data, data collection and structure refinement details are summarized in Table 4 ▸. The hydrogen atoms were placed in calculated positions and refined as riding models with fixed isotropic displacement parameters [*U*_iso_(H) = 1.5*U*_eq_(O), 1.5*U*_eq_(C) for the CH_3_-groups and 1.2*U*_eq_(C) for the other groups]. The remaining positive and negative residual electron densities are both located near the selenium atom.

## Supplementary Material

Crystal structure: contains datablock(s) I. DOI: 10.1107/S205698902500115X/tx2093sup1.cif

Structure factors: contains datablock(s) I. DOI: 10.1107/S205698902500115X/tx2093Isup2.hkl

Supporting information file. DOI: 10.1107/S205698902500115X/tx2093Isup3.cml

CCDC reference: 2300276

Additional supporting information:  crystallographic information; 3D view; checkCIF report

## Figures and Tables

**Table 1:** Hydrogen-bond geometry (Å, °)

*D*—H⋯*A*	*D*—H	H⋯*A*	*D*⋯*A*	*D*—H⋯*A*
C2—H2⋯Cl1	0.95	2.58	3.270 (3)	129
C2—H2⋯N3^i^	0.95	2.62	3.285 (3)	127
C5—H5⋯Cl1^ii^	0.95	2.66	3.316 (3)	127
C10—H10⋯N3^iii^	0.95	2.52	3.154 (4)	124

Symmetry codes: (i) 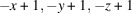; (ii) 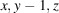; (iii) 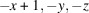.

**Table 2:** Inter­atomic contacts (Å)

Contact	Distance	Symmetry operation
H2⋯Cl1	2.58	*x*, *y*, *z*
Se1⋯S1	3.66	*x*, 1 + *y*, *z*
C2⋯Se1	3.56	−*x*, 1 − *y*, 1 − *z*
H2⋯N3	2.62	1 − *x*, 1 − *y*, 1 − *z*
S1⋯Se1	3.66	*x*, −1 + *y*, *z*
C10⋯C9	3.41	−*x*, −*y*, −*z*
N1⋯Cl1	3.39	−*x*, 1 − *y*, 1 − *z*
N3⋯H3	2.66	*x*, *y*, −1 + *z*
H10⋯N3	2.52	1 − *x*, −*y*, −*z*
H5⋯Cl1	2.66	*x*, −1 + *y*, *z*
H8⋯C4	3.03	−*x*, −*y*, 1 − *z*
H4⋯C10	2.97	1 − *x*, −*y*, 1 − *z*

**Table 3:** Values of QTAIM parameters at the bond-critical points (3, −1), corresponding to chalcogen bonds Se⋯Cl and Se⋯S in the X-ray structure ρ(**r**) = density of all electrons, ∇^2^ρ(**r**) = Laplacian of electron density, λ_2_ = eigenvalue, [*H*_b_] = energy density, *V*(**r**) = potential energy density, *G*(**r**) = Lagrangian kinetic energy, ELF (a.u.) = electron localization function and *E*_int_ = estimated strength for these inter­actions (kcal mol^−1^)

Contact^*a*^	Se⋯Cl, 2.843 Å, 78% vdW sum	Se⋯S, 3.656 Å, 99% vdW sum
ρ(**r**)	0.030	0.006
∇^2^ρ(**r**)	0.067	0.020
λ_2_	−0.030	−0.006
*H*_b_	−0.001	0.001
*V*(**r**)	−0.019	−0.003
*G*(**r**)	0.018	0.004
ELF	0.178	0.024
*E*_int_	6.0	0.9

Note: (*a*) The van der Waals (vdW) radii for S, Se, and Cl atoms are 1.80, 1.90, and 1.75 Å, respectively (Bondi, 1966 ▸).

**Table 4:** Experimental details

Crystal data	
Chemical formula	C_11_H_6_N_3_SSe^+^·Cl^−^
*M*_r_	326.66
Crystal system, space group	Triclinic, *P* 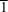
Temperature (K)	100
*a*, *b*, *c* (Å)	7.142 (3), 8.824 (4), 10.255 (5)
α, β, γ (°)	101.566 (13), 107.022 (14), 97.55 (1)
*V* (Å^3^)	592.8 (5)
*Z*	2
Radiation type	Mo *K*α
μ (mm^−1^)	3.55
Crystal size (mm)	0.60 × 0.40 × 0.10

Data collection	
Diffractometer	Bruker D8 Venture
Absorption correction	Multi-scan (*SADABS*; Krause *et al.*, 2015 ▸)
*T*_min_, *T*_max_	0.470, 0.746
No. of measured, independent and observed [*I* > 2σ(*I*)] reflections	7083, 3934, 3411
*R*_int_	0.035
(sin θ/λ)_max_ (Å^−1^)	0.755

Refinement	
*R*[*F*^2^ > 2σ(*F*^2^)], *wR*(*F*^2^), *S*	0.034, 0.081, 1.05
No. of reflections	3934
No. of parameters	154
H-atom treatment	H-atom parameters constrained
Δρ_max_, Δρ_min_ (e Å^−3^)	1.22, −0.74

Computer programs: *SAINT* (Bruker, 2019 ▸), *SHELXT* (Sheldrick, 2015*a* ▸), *SHELXL2018* (Sheldrick, 2015*b* ▸), *ORTEP-3 for Windows* (Farrugia, 2012 ▸) and *PLATON* (Spek, 2020 ▸).

## supplementary crystallographic information

### 3-(4-Cyanothiophen-3-yl)-[1,2,4]selenadiazolo[4,5-a]pyridin-4-ium chloride Crystal data

**Table:**

C_11_H_6_N_3_SSe^+^·Cl^−^	*Z* = 2
*M**_r_* = 326.66	*F*(000) = 320
Triclinic, *P*1	*D*_x_ = 1.830 Mg m^−^^3^
*a* = 7.142 (3) Å	Mo *K*α radiation, λ = 0.71073 Å
*b* = 8.824 (4) Å	Cell parameters from 5323 reflections
*c* = 10.255 (5) Å	θ = 2.4–32.5°
α = 101.566 (13)°	µ = 3.55 mm^−^^1^
β = 107.022 (14)°	*T* = 100 K
γ = 97.55 (1)°	Block, colourless
*V* = 592.8 (5) Å^3^	0.60 × 0.40 × 0.10 mm

### 3-(4-Cyanothiophen-3-yl)-[1,2,4]selenadiazolo[4,5-a]pyridin-4-ium chloride Data collection

**Table:**

Bruker D8 Venture diffractometer	3934 independent reflections
Radiation source: sealed X-ray tube	3411 reflections with *I* > 2σ(*I*)
Graphite monochromator	*R*_int_ = 0.035
Detector resolution: 5.6 pixels mm^-1^	θ_max_ = 32.4°, θ_min_ = 2.2°
ω scans	*h* = −10→8
Absorption correction: multi-scan (SADABS; Krause *et al.*, 2015)	*k* = −12→12
*T*_min_ = 0.470, *T*_max_ = 0.746	*l* = −15→14
7083 measured reflections	

### 3-(4-Cyanothiophen-3-yl)-[1,2,4]selenadiazolo[4,5-a]pyridin-4-ium chloride Refinement

**Table:**

Refinement on *F*^2^	0 restraints
Least-squares matrix: full	Hydrogen site location: inferred from neighbouring sites
*R*[*F*^2^ > 2σ(*F*^2^)] = 0.034	H-atom parameters constrained
*wR*(*F*^2^) = 0.081	*w* = 1/[σ^2^(*F*_o_^2^) + (0.0221*P*)^2^ + 0.1631*P*] where *P* = (*F*_o_^2^ + 2*F*_c_^2^)/3
*S* = 1.05	(Δ/σ)_max_ = 0.001
3934 reflections	Δρ_max_ = 1.22 e Å^−^^3^
154 parameters	Δρ_min_ = −0.74 e Å^−^^3^

### 3-(4-Cyanothiophen-3-yl)-[1,2,4]selenadiazolo[4,5-a]pyridin-4-ium chloride Special details

**Table:**

Geometry. All esds (except the esd in the dihedral angle between two l.s. planes) are estimated using the full covariance matrix. The cell esds are taken into account individually in the estimation of esds in distances, angles and torsion angles; correlations between esds in cell parameters are only used when they are defined by crystal symmetry. An approximate (isotropic) treatment of cell esds is used for estimating esds involving l.s. planes.

### 3-(4-Cyanothiophen-3-yl)-[1,2,4]selenadiazolo[4,5-a]pyridin-4-ium chloride Fractional atomic coordinates and isotropic or equivalent isotropic displacement parameters (Å^2^)

**Table:**

	*x*	*y*	*z*	*U*_iso_*/*U*_eq_	
Se1	0.19350 (3)	0.53066 (2)	0.38485 (2)	0.01314 (6)	
S1	0.06346 (8)	−0.22986 (6)	0.13180 (6)	0.02063 (12)	
N1	0.2726 (2)	0.27311 (19)	0.46985 (18)	0.0114 (3)	
N2	0.1683 (3)	0.3416 (2)	0.25768 (19)	0.0151 (3)	
N3	0.4496 (3)	0.2362 (2)	0.0365 (2)	0.0213 (4)	
C1	0.2707 (3)	0.4265 (2)	0.5271 (2)	0.0117 (3)	
C2	0.3256 (3)	0.4828 (2)	0.6731 (2)	0.0143 (4)	
H2	0.323858	0.589089	0.713832	0.017*	
C3	0.3824 (3)	0.3817 (3)	0.7575 (2)	0.0163 (4)	
H3	0.419393	0.418052	0.856995	0.020*	
C4	0.3855 (3)	0.2246 (2)	0.6958 (2)	0.0148 (4)	
H4	0.425057	0.154979	0.753641	0.018*	
C5	0.3317 (3)	0.1726 (2)	0.5530 (2)	0.0144 (4)	
H5	0.335128	0.067141	0.511177	0.017*	
C6	0.2087 (3)	0.2315 (2)	0.3197 (2)	0.0139 (4)	
C7	0.1862 (3)	0.0675 (2)	0.2395 (2)	0.0144 (4)	
C8	0.0845 (3)	−0.0641 (3)	0.2593 (2)	0.0186 (4)	
H8	0.032150	−0.063504	0.334549	0.022*	
C9	0.2484 (3)	0.0300 (2)	0.1180 (2)	0.0148 (4)	
C10	0.1904 (3)	−0.1269 (3)	0.0492 (2)	0.0191 (4)	
H10	0.217413	−0.172478	−0.033747	0.023*	
C11	0.3608 (3)	0.1445 (3)	0.0728 (2)	0.0176 (4)	
Cl1	0.22732 (8)	0.81017 (6)	0.59166 (6)	0.02006 (11)	

### 3-(4-Cyanothiophen-3-yl)-[1,2,4]selenadiazolo[4,5-a]pyridin-4-ium chloride Atomic displacement parameters (Å^2^)

**Table:**

	*U*^11^	*U*^22^	*U*^33^	*U*^12^	*U*^13^	*U*^23^
Se1	0.01299 (10)	0.01398 (11)	0.01459 (10)	0.00329 (7)	0.00538 (8)	0.00667 (7)
S1	0.0196 (3)	0.0153 (2)	0.0223 (3)	0.0013 (2)	0.0030 (2)	0.0015 (2)
N1	0.0108 (7)	0.0118 (8)	0.0116 (8)	0.0006 (6)	0.0042 (6)	0.0031 (6)
N2	0.0143 (8)	0.0171 (8)	0.0139 (8)	0.0031 (6)	0.0044 (7)	0.0045 (7)
N3	0.0198 (9)	0.0275 (10)	0.0181 (9)	0.0049 (8)	0.0082 (8)	0.0057 (8)
C1	0.0108 (9)	0.0123 (9)	0.0132 (9)	0.0022 (7)	0.0051 (7)	0.0038 (7)
C2	0.0144 (9)	0.0138 (9)	0.0149 (9)	0.0016 (7)	0.0053 (8)	0.0038 (7)
C3	0.0152 (10)	0.0191 (10)	0.0149 (10)	0.0015 (8)	0.0047 (8)	0.0063 (8)
C4	0.0142 (9)	0.0156 (9)	0.0144 (9)	0.0012 (7)	0.0036 (8)	0.0063 (8)
C5	0.0135 (9)	0.0114 (9)	0.0188 (10)	0.0017 (7)	0.0052 (8)	0.0058 (7)
C6	0.0128 (9)	0.0159 (9)	0.0129 (9)	0.0011 (7)	0.0051 (7)	0.0032 (7)
C7	0.0134 (9)	0.0161 (9)	0.0122 (9)	0.0024 (7)	0.0027 (7)	0.0029 (7)
C8	0.0197 (10)	0.0177 (10)	0.0166 (10)	0.0000 (8)	0.0057 (8)	0.0030 (8)
C9	0.0123 (9)	0.0180 (10)	0.0131 (9)	0.0045 (7)	0.0031 (7)	0.0027 (7)
C10	0.0148 (10)	0.0239 (11)	0.0167 (10)	0.0056 (8)	0.0039 (8)	0.0013 (8)
C11	0.0171 (10)	0.0232 (11)	0.0122 (9)	0.0064 (8)	0.0048 (8)	0.0022 (8)
Cl1	0.0238 (3)	0.0131 (2)	0.0243 (3)	0.00350 (19)	0.0087 (2)	0.0060 (2)

### 3-(4-Cyanothiophen-3-yl)-[1,2,4]selenadiazolo[4,5-a]pyridin-4-ium chloride Geometric parameters (Å, º)

**Table:**

Se1—N2	1.8511 (19)	C3—C4	1.410 (3)
Se1—C1	1.865 (2)	C3—H3	0.9500
S1—C10	1.705 (2)	C4—C5	1.362 (3)
S1—C8	1.712 (2)	C4—H4	0.9500
N1—C1	1.366 (3)	C5—H5	0.9500
N1—C5	1.372 (3)	C6—C7	1.477 (3)
N1—C6	1.426 (3)	C7—C8	1.370 (3)
N2—C6	1.283 (3)	C7—C9	1.434 (3)
N3—C11	1.148 (3)	C8—H8	0.9500
C1—C2	1.397 (3)	C9—C10	1.368 (3)
C2—C3	1.381 (3)	C9—C11	1.440 (3)
C2—H2	0.9500	C10—H10	0.9500
			
N2—Se1—C1	87.24 (9)	C4—C5—H5	120.1
C10—S1—C8	92.64 (11)	N1—C5—H5	120.1
C1—N1—C5	121.45 (18)	N2—C6—N1	117.21 (19)
C1—N1—C6	113.64 (17)	N2—C6—C7	121.7 (2)
C5—N1—C6	124.91 (18)	N1—C6—C7	121.10 (18)
C6—N2—Se1	111.81 (15)	C8—C7—C9	111.61 (19)
N1—C1—C2	119.91 (19)	C8—C7—C6	125.30 (19)
N1—C1—Se1	110.02 (15)	C9—C7—C6	122.64 (18)
C2—C1—Se1	130.06 (16)	C7—C8—S1	111.62 (17)
C3—C2—C1	119.0 (2)	C7—C8—H8	124.2
C3—C2—H2	120.5	S1—C8—H8	124.2
C1—C2—H2	120.5	C10—C9—C7	112.93 (19)
C2—C3—C4	119.9 (2)	C10—C9—C11	123.21 (19)
C2—C3—H3	120.0	C7—C9—C11	123.85 (19)
C4—C3—H3	120.0	C9—C10—S1	111.19 (17)
C5—C4—C3	119.9 (2)	C9—C10—H10	124.4
C5—C4—H4	120.0	S1—C10—H10	124.4
C3—C4—H4	120.0	N3—C11—C9	179.7 (3)
C4—C5—N1	119.78 (19)		
			
C1—Se1—N2—C6	1.08 (15)	C5—N1—C6—N2	−176.41 (18)
C5—N1—C1—C2	−1.5 (3)	C1—N1—C6—C7	−175.22 (17)
C6—N1—C1—C2	178.63 (17)	C5—N1—C6—C7	4.9 (3)
C5—N1—C1—Se1	177.54 (14)	N2—C6—C7—C8	−129.2 (2)
C6—N1—C1—Se1	−2.4 (2)	N1—C6—C7—C8	49.5 (3)
N2—Se1—C1—N1	0.80 (14)	N2—C6—C7—C9	42.5 (3)
N2—Se1—C1—C2	179.7 (2)	N1—C6—C7—C9	−138.8 (2)
N1—C1—C2—C3	0.5 (3)	C9—C7—C8—S1	−0.5 (2)
Se1—C1—C2—C3	−178.32 (15)	C6—C7—C8—S1	172.03 (17)
C1—C2—C3—C4	0.4 (3)	C10—S1—C8—C7	0.06 (18)
C2—C3—C4—C5	−0.2 (3)	C8—C7—C9—C10	0.7 (3)
C3—C4—C5—N1	−0.7 (3)	C6—C7—C9—C10	−171.97 (19)
C1—N1—C5—C4	1.6 (3)	C8—C7—C9—C11	−179.0 (2)
C6—N1—C5—C4	−178.50 (18)	C6—C7—C9—C11	8.3 (3)
Se1—N2—C6—N1	−2.7 (2)	C7—C9—C10—S1	−0.7 (2)
Se1—N2—C6—C7	175.96 (15)	C11—C9—C10—S1	179.05 (17)
C1—N1—C6—N2	3.5 (3)	C8—S1—C10—C9	0.37 (18)

### 3-(4-Cyanothiophen-3-yl)-[1,2,4]selenadiazolo[4,5-a]pyridin-4-ium chloride Hydrogen-bond geometry (Å, º)

**Table:**

*D*—H···*A*	*D*—H	H···*A*	*D*···*A*	*D*—H···*A*
C2—H2···Cl1	0.95	2.58	3.270 (3)	129
C2—H2···N3^i^	0.95	2.62	3.285 (3)	127
C5—H5···Cl1^ii^	0.95	2.66	3.316 (3)	127
C10—H10···N3^iii^	0.95	2.52	3.154 (4)	124

Symmetry codes: (i) −*x*+1, −*y*+1, −*z*+1; (ii) *x*, *y*−1, *z*; (iii) −*x*+1, −*y*, −*z*.
